# Chemical Interactions of Deicing Salts with Concrete Pastes Containing Slag Cement

**DOI:** 10.3390/ma18173962

**Published:** 2025-08-24

**Authors:** Mohsen Torabi, Peter C. Taylor

**Affiliations:** 1 Department of Civil, Construction & Environmental Engineering, Iowa State University, Ames, IA 50011, USA; 2 National Concrete Pavement Tech Center, Ames, IA 50010, USA

**Keywords:** slag cement, deicing salts, concrete durability, reaction mechanism, deicing salt corrosion, slag concrete

## Abstract

Chloride-based deicing salt solutions have been contacted with concrete pastes containing slag cement at different conditions, such as slag replacement (20–80%), type (CaCl_2_, MgCl_2_, NaCl), and concentration (1 M–5 M) of the deicing salt, as well as temperature (ambient & −18 °C), and the extent of their reactions have been studied using XRD and ICP-OES. Also, solubility of Friedel salt (FS) has been measured in different types and concentrations of deicing salt solutions. It has been observed that the chemical deterioration arising from the formation and then dissolution of FS is more significant than the damage caused by the formation and expansion of oxychlorides in the pastes containing slag. While calcium oxychloride in its dried form can linger inside the paste for a long time, FS undergoes incongruent dissolution in CaCl_2_ and MgCl_2_ solutions and leaves the system. Presence of higher levels of AFm phases in pastes containing slag will further underscore this phenomenon. The extent of this chemical deterioration is relatively lower in NaCl solutions. Also, it was found that the nature of the chemical interaction changes with the concentration of the salt, as some disappeared phases might reappear and then disappear again. Using XRD and ICP-OES, this study provides a mechanistic understanding of salt-induced chemical deterioration in slag cement pastes by identifying phase-specific vulnerabilities and tracking the formation, transformation, and dissolution of key phases, such as Friedel’s salt and calcium oxychloride; additionally, the influence of various parameters have been studied, and chemical mechanisms have been proposed.

## 1. Introduction

Utilization of supplementary cementitious materials (SCMs) can provide a practical solution to reducing environmental emissions from the concrete construction industry. Slag cement is a byproduct of the iron making process in blast furnaces, which exhibits cementitious properties if rapidly quenched at the exit of the blast furnace. Incorporation of slag into concrete can not only reduce cement usage but also can bring gains to the mechanical [1,2,3] and chemical resistance properties of the resulting concrete [4,5,6]. Despite these benefits, there are a few drawbacks in using slags, including their perceived reduced chemical resistance against deicing salts that results in salt scaling in cold climates. This mechanism is normally limited to the concrete surface but can affect ride and later durability of the slab [7].

Since deicing salts are only used in cold climates, scaling is concomitant with freeze/thaw (F/T) damage and a distinction between them is necessary for better understanding their mechanisms and developing remedies. Concrete can adsorb water and when the ambient temperature falls sufficiently, this absorbed water can freeze and expand and therefore induce pressure in the capillaries of the concrete. If this pressure exceeds the tensile strength of the concrete matrix, it will cause dilation [8,9] and eventually will lead to rupture of the sample [10,11]. While presence of some ions might accelerate the water saturation of the concrete and therefore accelerate F/T damage, it is appropriate to consider that F/T is a physical damage mechanism, which is usually also prevented physically via incorporation of some air bubbles into the concrete. On the other hand, the attack of deicing salts to concrete is a chemical phenomenon that depends on the type and concentration of the deicing salt species as well as the phases inside the concrete [12,13].

One of the mechanisms for the initiation and propagation of salt scaling is formation of oxychlorides that are complexes of the attacking chloride species and the hydroxide phase in concrete. For instance, calcium oxychloride (COX), with the structure $3Ca{\left(OH\right)}_{2}•Ca{Cl}_{2}•15H_{2}O$, or its dried form $CaO•Ca{Cl}_{2}•2H_{2}O$, are complexes of calcium hydroxide and calcium chloride; similarly, magnesium oxychloride (MOX), with a formula of $Mg{\left(OH\right)}_{2}\cdot 5Mg{Cl}_{2}\cdot 8H_{2}O$, is a complex of magnesium hydroxide and magnesium chloride. Oxychlorides can imbibe large amounts of water and expand significantly [14], causing cracks in the matrix. Therefore, oxychlorides are a kind of malady that form chemically but damage the concrete physically via expansion. Formation of calcium oxychlorides has been verified in the lab [15,16,17].

Since utilization of SCMs (supplementary cementitious materials) results in the reduction in portlandite, both due to the pozzolanic reaction as well as dilution, some researchers have concluded that SCMs can reduce salt scaling damage due to a lower amount of portlandite present to form oxychlorides [18,19]. However, other studies have observed higher levels of such damage in concretes containing slag [20,21,22]. In addition, other studies have reported higher salt scaling in concretes containing slag [23,24,25]. These observations suggest that the chemical attributes of the interaction between deicing salts and the concretes containing slag might not be limited just to oxychlorides [19,26].

Another influencing mechanism can be carbonation, as this process has been considered to enhance salt scaling [27,28]. It is proposed that during carbonation, some metastable forms of calcium carbonate such as vaterite that have some solubility in chloride solutions might form [29,30], and this might be the reason for this vulnerability [31]. Nonetheless, there might be some skepticism regarding this means of concrete deterioration. First, concretes containing slag are assumed to have lower levels of portlandite and therefore are less prone to the formation of calcium carbonate in any of its crystalline phases; thence, the resulting damage is assumed to be lower. The second reason is that at its best, this mechanism can provide an external reason for the related concrete corrosion, since it is not directly related to the attacking deicing salt species or the phases inside the concrete.

On the other hand, deicing salts that introduce significant quantities of chloride ions to the system have sufficient potential to cause modifications in the concrete matrix. Moreover, the cation accompanying the chloride ions might also have substantial influence on the extent of this type of chemical deterioration [32]. Chloride ions can not only become adsorbed on the surface of the reaction products, but also, they can alter their phase via ion exchange [33]. Friedel salt (3CaO • Al_2_O_3_ • CaCl_2_ • 10H_2_O) is probably the major phase that is formed as a result of adsorption of chloride into ${\left[{Ca}_{2}Al{\left(OH\right)}_{6}•2H_{2}O\right]}^{+}$ layers or ion exchange of chloride with AFm phases [34]. The AFm phases (alumina, ferric oxide, monosulfate) are a group of hydrated calcium aluminate compounds that form during the hydration of cement. Phases like monosulfoaluminate, monocarboaluminate, etc., belong to this group. The chloride ions connect to the ${\left[{Ca}_{2}Al{\left(OH\right)}_{6}•2H_{2}O\right]}^{+}$ via hydrogen bonding in the FS (Friedel salt) crystal structure [35] and form a phase that is relatively stable in the concrete paste matrix that has a high pH; therefore, this phase (FS) plays an important role in the chloride binding capacity of the concrete pastes. Consequently, phases with a layered double-hydroxide (LDH) structure have a prominent role in chloride binding [36] through their conversion to FS (except hydrotalcite). However, there are some caveats in this field, as the researchers studying the chloride binding capacity of slag-containing concretes have come to different observations [23,37]. However, regardless of this, formation and stability of the FS are necessary parts of the interactions between the deicing salts and concrete pastes containing slag. This becomes even more pronounced in the concrete pastes containing SCMs, including slag, as the AFm phases which form a precursor for FS concentrate in these systems.

While most of the studies performed on the interaction between deicing salts and concretes containing slags have considered the influence of the factors such as water-to-binder ratio, curing temperature, or slag replacement [25,38,39,40,41], there is still a need for a better understanding of the reaction mechanisms from a chemical perspective. This manuscript aims at providing this insight by studying the influence of the deicing salt type, concentration, implementation temperature, and replacement levels on the extent of the damage caused by deicing salts. Also, stability of the related phases formed during the reaction between deicing salts and concrete pastes containing slag will be studied.

This study is designed to go beyond conventional performance-based assessments by delivering a detailed chemical characterization of how chloride-based deicing salts interact with slag-containing cement pastes. Through controlled experiments varying salt type, concentration, temperature, and slag replacement level, this work aims to isolate and study the mechanisms of degradation, with particular focus on the formation, transformation, and dissolution of key phases such as Friedel’s salt and calcium oxychloride and their possible decomposition. By integrating XRD and ICP-OES analysis, this study provides a mechanistic understanding of salt-induced chemical deterioration, identifies phase-specific vulnerabilities of slag-blended systems, and offers new insights into how these reactions evolve under real-world exposure conditions. The outcomes are expected to fill critical gaps in current knowledge and offer a scientifically grounded framework to guide material selection and durability design for concrete infrastructure exposed to aggressive winter environments.

## 2. Materials and Methods

Reagent-grade aluminum hydroxide and sodium hydroxide were purchased from Millipore Sigma, and magnesium chloride, calcium chloride, sodium chloride, nitric acid, and isopropanol were procured from Fisher Scientific. The elemental analysis for the used cement and slag are in Table 1 below. Elemental analysis was performed using XRF.

Friedel salt was synthesized by reacting calcium chloride with sodium aluminate, similar to the approach reported in the literature [42]. Sodium aluminate was synthesized by dissolving aluminum hydroxide in a sodium hydroxide solution. Excess calcium chloride was used in the reaction to ensure consumption of all sodium aluminate. The temperature of the system was controlled, and the solution was constantly mixed using magnetic stirrers. The suspension was filtered and washed several times to get rid of any remaining calcium chloride or sodium hydroxide in the system. Then, the product was dried in an oven overnight and kept in sealed containers. The purity of the synthesized Friedel salt was ascertained using XRD.

Paste samples were prepared with various slag replacements from 0% wt to 100% wt at 20% wt increments (Table 2). The water-to-binder ratio was kept at 0.4 wt for all the samples. After mixing, the paste was poured into polyethylene cylinders, and their caps were sealed till the time of cutting. After 120 days of hydration, slices were cut, fine ground, reacted with deicing salts, and kept inside glass vials with sealed caps to eliminate carbonation from air. The samples were kept in contact with the deicing salt solutions (Table 3) for 15 days and then tested. Some samples were kept inside a freezer at −18 °C for three weeks before testing to assess the effects of reduced temperatures. pH measurement was performed using a Metler Toledo benchtop pH meter.

X-ray diffraction of tested paste samples was performed using a Rigaku Smartlab XRD (Tokyo, Japan), and all the experiments were performed using a parallel beam configuration ranging from 7° to 60° with a step size of 1°/min. ICP-OES was performed using a Shimadzu ICP-OES 9810 device (Kyoto, Japan) on samples diluted with 0.2 M nitric acid. The analytical wavelength used for calcium was 220.861 nm. This range of diffraction angles will contain the major diffraction peaks of all phases under study with their strongest intensities; therefore, there was no need to study the very weak and strongly overlapped peaks that happen after 60°. Reference standards can be found in ASTM C1365 [43], ASTM C114 [44], and ASTM C1875 [45].

## 3. Results

### 3.1. Phase Identification Using X-Ray Diffraction

**Figure 1:**
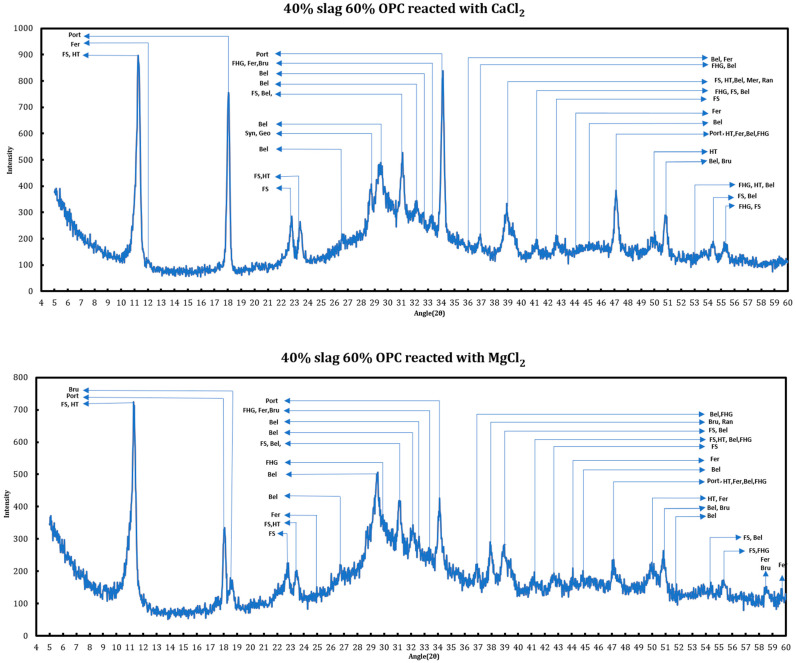
Phase identification of the mature slag + cement samples contacted with deicing salt solutions. FS: Friedel salt, HT: hydrotalcite, Port: portlandite, Bru: brucite, Fer: ferrite, Bel: belite, Syn: syngenite, Geo: gorgeyite, FHS: siliceous hydrogarnet.

X-ray diffraction has been utilized for phase identification of the cement + slag pastes reacted with 1 M CaCl_2_ and 1 M MgCl_2_. The main changes in the appearing phases are related to the disappearance of ettringite, conversion of AFm phases to Friedel salt and later its dissolution, disappearance of portlandite, and the formation of oxychlorides. As examples, the phase identification results for a cement + slag paste with a slag replacement level of 40% and hydrated for more than 120 days that has been reacted with 1 M CaCl_2_ and 1 M MgCl_2_ have been provided in the Figure 1.

### 3.2. Influence of the Type of Deicing Salt

**Figure 2:**
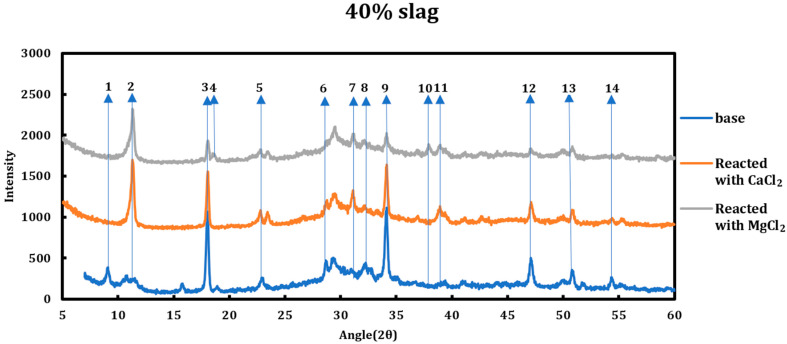
XRD spectra related to the 40% slag + 60% cement sample as well as the cases when the sample was reacted with 1 M CaCl_2_ and 1 M MgCl_2_. The reflections that showed change in the spectra are enumerated.

**Figure 3:**
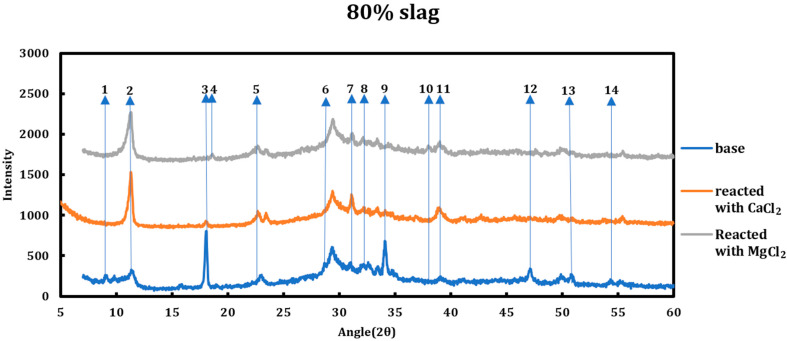
XRD spectra related to the 80% slag + 20% cement sample as well as the cases when the sample was reacted with 1 M CaCl_2_ and 1 M MgCl_2_. The reflections that showed change in the spectra are enumerated.

The type of deicing salt has a significant effect on the type and the extent of the chemical deterioration taking place. To better understand this issue, 1 M calcium chloride and 1 M magnesium chloride were contacted with two slag + cement samples with slag replacements of 40% and 80% and then characterized using XRD (Figure 2 and Figure 3). The resulting spectra have been compared to the spectra of the samples before the reaction. All the reflections that displayed changes in the original spectrum are enumerated and explained hereafter.

The chemical interactions between various phases in the system are understood through studying every single reflection peak in the corresponding XRD spectrum. Any reduction in the relative intensity of a peak, if accompanied by other related peaks, might indicate reductions in the concentration of the related phase inside the system. Accordingly, disappearance of a group of peaks related to a phase can suggest total consumption of that phase.

The phenomena observed during the addition of $Ca{Cl}_{2}/Mg{Cl}_{2}$ solutions to the slag + cement paste can be summarized as follows:

- Conversion of AFm phases to Friedel salt: formation of Friedel salt is vivid through its reflections at 11.36°, 22.84°, 23.53°, 31.23°, 39.04°, and 55.57°. The chloride ion liberated from deicing salt solutions replaces the host ion between double-hydroxide layers of AFm phases and forms Friedel salt.
- Decomposition of ettringite: ettringite is decomposed when the slag + cement paste is contacted with magnesium/calcium chloride solutions based on the suppression of signals at 9.08°, 15.77°, 18.86°, 22.89°, 32.23°, and 40.81°. The ions liberated from the solution via ettringite decomposition might contribute to the formation of Friedel salt.
- Consumption of portlandite: portlandite is consumed in both cases as the intensity of its main signals (18.18°, 28.73°, 34.22°, 47.39°, 50.90°, 54.49°) diminishes and this case is even more pronounced in the case of magnesium chloride solution.
- Formation of brucite in the case of $Mg{Cl}_{2}$: brucite is only formed in the case of magnesium chloride solution clear from its reflections at 18.60°, 38.05°, 50.89°, and 58.72°.

Location of the changes described previously are enumerated on the XRD graphs (Figure 2 and Figure 3) and they are explained as follows:

1. Dissolution of ettringite (it is thought that its constituents are converted to the Friedel salt).
2. Friedel salt’s main signal occurring on 11.36° is related to its (006) reflection. This signal overlaps with the (003) reflection of hydrotalcite (R-3 M).
3. Depletion of portlandite: this phenomenon is more vividly observed in the case of magnesium chloride solution.
4. Brucite formation, which happens in the case of magnesium chloride solution.
5. Friedel salt formation. FS has two peaks at 22.8° (0012) and 23.5° (018) (the second one overlaps with one of hydrotalcite signals).
6. Dissolution of syngenite and gorgeyite. This issue is only witnessed in the case of magnesium chloride solutions.
7. Friedel salt formation. FS (110) reflection appears at 31.22°.
8. Conversion of ettringite and AFm phases to Friedel salt. Signals at 32.12° and 32.5° are superpositions of signals from ettringite and other AFm phases that are converted to Friedel salt and their signals have lessened.
9. Depletion of portlandite: this phenomenon is more vividly observed in the case of magnesium chloride solution.
10. Brucite formation, which happens in the case of magnesium chloride solution.
11. Friedel salt formation. FS (1112 and 208) signals appear at 31.2° and 39.47°, respectively, which overlap and form a stronger signal.
12. Depletion of portlandite: this phenomenon is more vividly observed in the case of magnesium chloride solution.
13. Depletion of portlandite and conversion of AFm phases to Friedel salt. The signal at 54.3° is a superposition of other phases, mainly monocarboaluminate and hydrocalumite. Both these phases are converted to Friedel salt and their signals diminish.
14. Same as 13.

**Figure 4:**
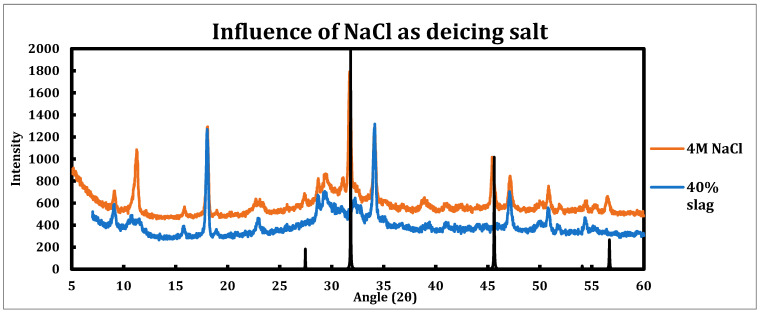
XRD spectra of 40% slag + 60% cement sample as well as when it is contacted with 4 M sodium chloride solution. The black spectrum is related to sodium chloride, as it has been crystallized as the paste sample was dried.

Similar experiments have been performed on the sample contacted with sodium chloride solutions (Figure 4). Since the sodium chloride solutions used for deicing can have concentrations as high as 25% [46], a 4 M NaCl solution has been chosen for this experiment to resemble the field conditions more. The sample being tested had a slag replacement of 40%.

Influence of sodium chloride as a deicing salt is as follows:

1. Sodium chloride is much less destructive in comparison to calcium/magnesium chloride.
2. Ettringite is clearly in place and is not converted by sodium chloride, as the reflections at 9.08° (100), 15.77° (110), 18.86° (10$\bar{4}$), 22.9° (11$\bar{4}$), and 25.6° (212) are clearly in place and distinguishable.
3. AFm phases (monosulfoaluminate, hydrocalumite, hemicarboaluminate, and monocarboaluminate) are converted to Friedel salt, which is explicit by its strong peak at 11.36° and related to (006) reflection.
4. The peaks observed at 27.47°, 31.82°, 45.63°, and 56.69° are related to the sodium chloride crystals.

### 3.3. Influence of Deicing Salt Concentration

**Figure 5:**
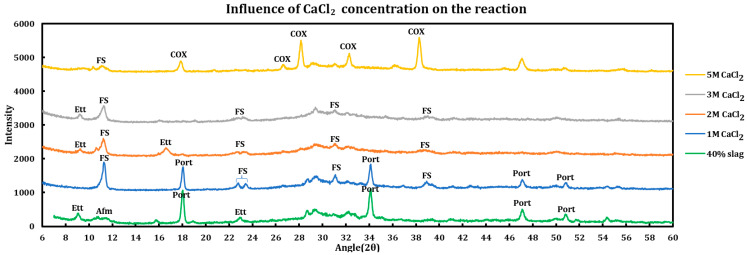
Influence of CaCl_2_ concentration on a 40% slag + 60% cement sample.

**Figure 6:**
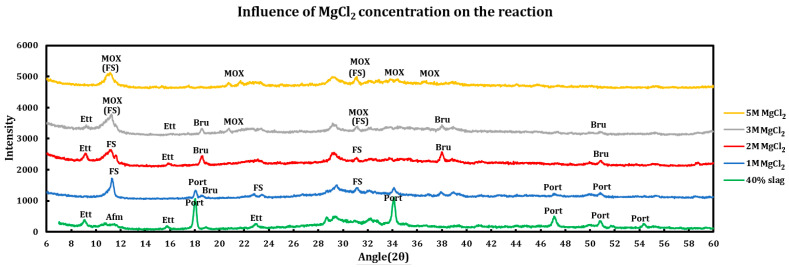
Influence of MgCl_2_ concentration on a 40% slag + 60% cement sample.

**Figure 7:**
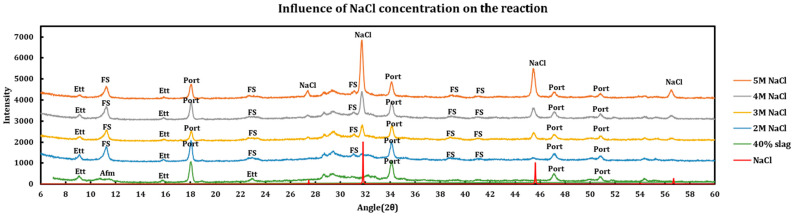
Influence of NaCl concentration on a 40% slag + 60% cement sample.

Influence of the salt concentrations on the extent of the chemical interaction was explored by contacting slag + cement paste with different concentrations of the deicing salt solutions. This experiment has been performed using a 40% slag + 60% cement with 1 M, 2 M, 3 M, and 5 M of calcium chloride (Figure 5), as well as magnesium chloride (Figure 6). Similar experiments have been run using sodium chloride at concentrations of 2 M, 3 M, 4 M, and 5 M (Figure 7).

#### 3.3.1. Calcium Chloride

The behavior of the system changes as the concentration of calcium chloride increases. While ettringite decomposes at 1 M CaCl_2_, it forms again at 2 M and 3 M but again vanishes from the system at the 5 M concentration. The presence of calcium oxychloride is explicit at a higher concentration (5 M).

Details of the phase changes due to an increase in CaCl_2_ concentration according to XRD spectra are as follows:

- At 1 M CaCl_2_, all AFm phases are converted to Friedel salt and all ettringite has undergone decomposition, products of which have probably contributed to the formation of more FS. Part of the portlandite has also been dissolved in the acidic environment. Decomposition of ettringite must increase the concentration of sulfate ions in the system. Since gypsum has not been observed among the reaction products, the following route is proposed for ettringite decomposition and FS formation:

$$Ettringite\;decomposition:\;2{\left[{Ca}_{3}\cdot Al{\left(OH\right)}_{6}\right]}^{+3}•\left(3SO_{4}^{-2}\right)•26H_{2}O⇌2{\left[{Ca}_{2}•Al{\left(OH\right)}_{6}\right]}^{+}+2{Ca}^{+2}+3\left(SO_{4}^{-2}\right)+26H_{2}O.$$

$$Friedel\;salt\;formation:\;{2Cl}^{-}+2{\left[{Ca}_{2}•Al{\left(OH\right)}_{6}\right]}^{+}•({OH}^{-},{{CO}_{3}}^{-2},SO_{4}^{-2})\cdot 6H_{2}O\to 2{\left[{Ca}_{2}•Al{\left(OH\right)}_{6}\right]}^{+}\cdot 2{Cl}^{-}\cdot 2H_{2}O+({OH}^{-},{{CO}_{3}}^{-2},SO_{4}^{-2})+4H_{2}O\;{2Cl}^{-}+2{\left[{Ca}_{2}•Al{\left(OH\right)}_{6}\right]}^{+}+2H_{2}O\to 2{\left[{Ca}_{2}•Al{\left(OH\right)}_{6}\right]}^{+}•2{Cl}^{-}•2H_{2}O$$

$$Portlandite\;depletion:\;Ca{\left(OH\right)}_{2}+2H^{+}⟶{Ca}^{+2}+2H_{2}O$$

- At 2 M CaCl_2_, intensity of Friedel salt has decreased, which can be due to its decomposition in acidic environments. FS can be decomposed either to its building blocks including calcium aluminum hydroxide ion layers and chloride, or it can form calcium ion, aluminum tetrahydroxide, calcium, and chloride ions through its decomposition. Formation of a new peak at 9.09° suggests resynthesis of ettringite. This can either be the result of reactions between the calcium aluminum hydroxide ion layers or the aluminum tetrahydroxide ions that are released due to the decomposition of FS or ettringite, with the sulfate and calcium ions that are abundant in the system. All portlandite in the system has been consumed, as its major reflections at 18.11° and 34.17° have completely disappeared.

$$FS\;dissolution:\;2{\left[{Ca}_{2}•Al{\left(OH\right)}_{6}\right]}^{+}•2{Cl}^{-}•2H_{2}O\to {2Cl}^{-}+{4Ca}^{+2}+2Al{\left(OH\right)}_{4}^{-}+4{\left(OH\right)}^{-}+2H_{2}O\;2{\left[{Ca}_{2}•Al{\left(OH\right)}_{6}\right]}^{+}•2{Cl}^{-}•2H_{2}O\to {2Cl}^{-}+2{\left[{Ca}_{2}•Al{\left(OH\right)}_{6}\right]}^{+}+2H_{2}O$$

$$Ettringite\;formation:\;2Al{\left(OH\right)}_{4}^{-}+6{Ca}^{+2}+4{\left(OH\right)}^{-}+3\left(SO_{4}^{-2}\right)+2{6H}_{2}O\to 2{\left[{Ca}_{3}•Al{\left(OH\right)}_{6}\right]}^{+3}•\left(3SO_{4}^{-2}\right)•26H_{2}O\;2{\left[{Ca}_{2}•Al{\left(OH\right)}_{6}\right]}^{+}+2{Ca}^{+2}+3\left(SO_{4}^{-2}\right)+26H_{2}O⇌2{\left[{Ca}_{3}•Al{\left(OH\right)}_{6}\right]}^{+3}•\left(3SO_{4}^{-2}\right)•26H_{2}O$$

$$Portlandite\;depletion:\;Ca{\left(OH\right)}_{2}+2H^{+}⟶{Ca}^{+2}+2H_{2}O$$

- 3 M CaCl_2_: based on lessening of the signal at 9.09°, it is observed that the ettringite has been decomposed since it is not stable in acidic conditions either. Signals of FS have also been decreased.

$$Ettringite\;decomposition:\;2{\left[{Ca}_{3}•Al{\left(OH\right)}_{6}\right]}^{+3}•\left(3SO_{4}^{-2}\right)•26H_{2}O⇌2{\left[{Ca}_{2}•Al{\left(OH\right)}_{6}\right]}^{+}+2{Ca}^{+2}+3\left(SO_{4}^{-2}\right)+26H_{2}O$$

$$FS\;dissolution:\;2{\left[{Ca}_{2}•Al{\left(OH\right)}_{6}\right]}^{+}•2{Cl}^{-}•2H_{2}O\to {2Cl}^{-}+{4Ca}^{+2}+2Al{\left(OH\right)}_{4}^{-}+4{\left(OH\right)}^{-}+2H_{2}O\;2{\left[{Ca}_{2}•Al{\left(OH\right)}_{6}\right]}^{+}•2{Cl}^{-}•2H_{2}O\to {2Cl}^{-}+2{\left[{Ca}_{2}•Al{\left(OH\right)}_{6}\right]}^{+}+2H_{2}O$$

- 5 M CaCl_2_: most of ettringite and a considerable amount of FS have been decomposed in this low pH environment. However, calcium oxychloride has been formed significantly, as can be observed by its vivid peaks at 18.04°, 26.87°, 28.34°, 32.56°, 36.52°, and 38.43°. The observed oxychloride is in the form of $CaO\cdot Ca{Cl}_{2}\cdot 2H_{2}O$, which is the dried form of the calcium oxychloride that has the formula of $Ca{Cl}_{2}\cdot 3CaO\cdot 15H_{2}O$ when it is wet. This result demonstrates that, despite the fact that FS decomposes in the low pH environment caused by a high concentration of calcium chloride solutions, COX in its dried form is relatively stable and compatible with this situation.

$$Calcium\;oxychloride\;formation:\;Ca{\left(OH\right)}_{2}+Ca{Cl}_{2}+15H_{2}O⟶3Ca{\left(OH\right)}_{2}•Ca{Cl}_{2}•15H_{2}O$$

$$Calcium\;oxychloride\;drying:\;3Ca{\left(OH\right)}_{2}•Ca{Cl}_{2}•15H_{2}O⟶CaO•Ca{Cl}_{2}•2H_{2}O+2CaO+16H_{2}O$$

$$Ettringite\;decomposition:\;2{\left[{Ca}_{3}•Al{\left(OH\right)}_{6}\right]}^{+3}•\left(3SO_{4}^{-2}\right)•26H_{2}O⇌2{\left[{Ca}_{2}•Al{\left(OH\right)}_{6}\right]}^{+}+2{Ca}^{+2}+3\left(SO_{4}^{-2}\right)+26H_{2}O$$

$$FS\;dissolution:\;2{\left[{Ca}_{2}•Al{\left(OH\right)}_{6}\right]}^{+}•2{Cl}^{-}•2H_{2}O\to {2Cl}^{-}+{4Ca}^{+2}+2Al{\left(OH\right)}_{4}^{-}+4{\left(OH\right)}^{-}+2H_{2}O\;2{\left[{Ca}_{2}•Al{\left(OH\right)}_{6}\right]}^{+}•2{Cl}^{-}•2H_{2}O\to {2Cl}^{-}+2{\left[{Ca}_{2}•Al{\left(OH\right)}_{6}\right]}^{+}+2H_{2}$$

In summary, the following phenomena have occurred in pastes contacted with calcium chloride solutions at different concentrations:

- Depletion of portlandite: as the concentration of CaCl_2_ increases, the depletion of portlandite increases as it becomes completely consumed.
- Disappearance of AFm phases: these phases have been converted to Friedel salt due to presence of chloride ion in the system released by the dissolution of calcium chloride.
- Decomposition, formation, and again decomposition of ettringite: this phase decomposed at 1 M but formed at 2 M and was later decomposed.
- Dissolution of Fridel salt: the intensity of this signal reduces continuously as the concentration of CaCl_2_ increases from 1 M to 5 M.
- Calcium oxychloride: this phase starts to appear at higher concentrations of CaCl_2_ and becomes clearly noticeable at 5 M. The peaks that we have observed in our experiment are related to the CaO • CaCl_2_ • 2H_2_O, which is the dried form of CaCl_2_ • 3CaO • 15H_2_O.
- Some peaks are partially related to the precipitation of the CaCl_2_ • 4H_2_O precipitated after drying of the sample.

#### 3.3.2. Magnesium Chloride

The influence of magnesium chloride concentrations on the extent of chemical reaction has also been studied, and the results are as follows:

- 1 M MgCl_2_: all AFm phases are converted to Friedel salt and ettringite has also decomposed. Portlandite is consumed mainly through conversion to brucite, which can be observed by the small peak shoulder on the right of portlandite (001) reflection happening at 18.1°.

$$Friedel\;salt\;formation:\;{2Cl}^{-}+2{\left[{Ca}_{2}•Al{\left(OH\right)}_{6}\right]}^{+}•\left({OH}^{-},{{CO}_{3}}^{-2},SO_{4}^{-2}\right)•6H_{2}O\to 2{\left[{Ca}_{2}•Al{\left(OH\right)}_{6}\right]}^{+}•2{Cl}^{-}•2H_{2}O+({OH}^{-},{{CO}_{3}}^{-2},SO_{4}^{-2})+4H_{2}O\;{2Cl}^{-}+2{\left[{Ca}_{2}•Al{\left(OH\right)}_{6}\right]}^{+}+2H_{2}O\to 2{\left[{Ca}_{2}•Al{\left(OH\right)}_{6}\right]}^{+}•2{Cl}^{-}•2H_{2}O$$

$$Ettringite\;decomposition:\;2{\left[{Ca}_{3}•Al{\left(OH\right)}_{6}\right]}^{+3}•\left(3SO_{4}^{-2}\right)•26H_{2}O⇌2{\left[{Ca}_{2}•Al{\left(OH\right)}_{6}\right]}^{+}+2{Ca}^{+2}+3\left(SO_{4}^{-2}\right)+26H_{2}O$$

$$Portlandite\;conversion:\;Ca{\left(OH\right)}_{2}+{Mg}^{+2}⟶Mg{\left(OH\right)}_{2}+{Ca}^{+2}$$

- 2 M MgCl_2_: portlandite is depleted (converted to brucite) and its signals disappeared. FS has dissolved incongruently and released its building blocks. Ettringite has been formed and magnesium oxychloride (MOX) shows signs of formation, as seen in the smaller peak shoulder on the right of the (006) reflection of Friedel salt.

$$FS\;dissolution:\;2{\left[{Ca}_{2}•Al{\left(OH\right)}_{6}\right]}^{+}•2{Cl}^{-}•2H_{2}O\to {2Cl}^{-}+{4Ca}^{+2}+2Al{\left(OH\right)}_{4}^{-}+4{\left(OH\right)}^{-}+2H_{2}O\;2{\left[{Ca}_{2}•Al{\left(OH\right)}_{6}\right]}^{+}•2{Cl}^{-}•2H_{2}O\to {2Cl}^{-}+2{\left[{Ca}_{2}•Al{\left(OH\right)}_{6}\right]}^{+}+2H_{2}O$$

$$Ettringite\;formation:\;2Al{\left(OH\right)}_{4}^{-}+6{Ca}^{+2}+4{\left(OH\right)}^{-}+3\left(SO_{4}^{-2}\right)+2{6H}_{2}O\to 2{\left[{Ca}_{3}•Al{\left(OH\right)}_{6}\right]}^{+3}•\left(3SO_{4}^{-2}\right)•26H_{2}O\;2{\left[{Ca}_{2}•Al{\left(OH\right)}_{6}\right]}^{+}+2{Ca}^{+2}+3\left(SO_{4}^{-2}\right)+26H_{2}O⇌2{\left[{Ca}_{3}•Al{\left(OH\right)}_{6}\right]}^{+3}•\left(3SO_{4}^{-2}\right)•26H_{2}O$$

$$Portlandite\;conversion:\;Ca{\left(OH\right)}_{2}+{Mg}^{+2}⟶Mg{\left(OH\right)}_{2}+{Ca}^{+2}$$

$$Magnesium\;oxychloride\;formation:\;xMg{\left(OH\right)}_{2}+yMg{Cl}_{2}+zH_{2}O⟶{Mg}_{x+y}•{\left(OH\right)}_{2x}•{Cl}_{2y}•{{(H}_{2}O)}_{z}\;Mg{\left(OH\right)}_{2}+5Mg{Cl}_{2}+8H_{2}O⟶2{Mg}_{3}•(OH)•{Cl}_{5}•{{(H}_{2}O)}_{8}$$

- 3 M MgCl_2_: ettringite undergoes decomposition mainly due to its incompatibility with lower pH environments. MOX forms the main signal at 11.8 and signal of the remaining FS is being added to it. Brucite signals are weakened mainly due to the reaction with magnesium chloride and formation of MOX.

$$FS\;dissolution:\;2{\left[{Ca}_{2}•Al{\left(OH\right)}_{6}\right]}^{+}•2{Cl}^{-}•2H_{2}O\to {2Cl}^{-}+{4Ca}^{+2}+2Al{\left(OH\right)}_{4}^{-}+4{\left(OH\right)}^{-}+2H_{2}O\;2{\left[{Ca}_{2}•Al{\left(OH\right)}_{6}\right]}^{+}•2{Cl}^{-}•2H_{2}O\to {2Cl}^{-}+2{\left[{Ca}_{2}•Al{\left(OH\right)}_{6}\right]}^{+}+2H_{2}O$$

$$Ettringite\;decomposition:\;2{\left[{Ca}_{3}•Al{\left(OH\right)}_{6}\right]}^{+3}•\left(3SO_{4}^{-2}\right)•26H_{2}O⇌2{\left[{Ca}_{2}•Al{\left(OH\right)}_{6}\right]}^{+}+2{Ca}^{+2}+3\left(SO_{4}^{-2}\right)+26H_{2}O$$

$$Magnesium\;oxychloride\;formation:\;xMg{\left(OH\right)}_{2}+yMg{Cl}_{2}+zH_{2}O⟶{Mg}_{x+y}•{\left(OH\right)}_{2x}•{Cl}_{2y}•{{(H}_{2}O)}_{z}\;Mg{\left(OH\right)}_{2}+5Mg{Cl}_{2}+8H_{2}O⟶2{Mg}_{3}•(OH)•{Cl}_{5}•{{(H}_{2}O)}_{8}$$

- 5 M MgCl_2_: MOX signals are clear and predominant and Friedel salt signals lie underneath the MOX signals. It is observed that similar to COX, MOX also seems to be stable in the current environment.

$$Magnesium\;oxychloride\;formation:\;xMg{\left(OH\right)}_{2}+yMg{Cl}_{2}+zH_{2}O⟶{Mg}_{x+y}•{\left(OH\right)}_{2x}•{Cl}_{2y}•{{(H}_{2}O)}_{z}\;Mg{\left(OH\right)}_{2}+5Mg{Cl}_{2}+8H_{2}O⟶2{Mg}_{3}•(OH)•{Cl}_{5}•{{(H}_{2}O)}_{8}$$

So, the phenomena taking place for these samples are as follows:

- Portlandite consumption: portlandite is almost completely consumed in the case of higher concentrations (2 M). In 1 M MgCl_2_, there are still reflections at 18.19° and 34.22° which are related to portlandite. However, at 2 M and 3 M, the first signal at 18.19° has shifted to 18.60°, and a new signal has appeared at 38.06°, both of which are related to brucite formation.
- Brucite: this phase is formed by conversion of portlandite but then it also disappears at 5 M MgCl_2_. This can be ascribed to the formation of magnesium oxychloride at the higher concentration of magnesium chloride.
- Magnesium oxychloride: while some studies have not observed this phase and claim that COX forms instead of it [19,47], this phase has been observed in this study. One of the issues in identification of this phase might be its overlap with the signals pertaining to FS. However, the major distinctive MOX signal that does not overlap with FS signals and lies at 21.5° is present in this system at the magnesium chloride concentration of 3 M and above.
- Calcium oxychloride: this phase is not observed in the samples.
- Magnesium chloride: the hydrated form (MgCl_2_•6H_2_O) starts to precipitate at a high concentration (5 M). This is mainly due to oversaturation of the sample at a high concentration and low temperature.

#### 3.3.3. Sodium Chloride

Effects of NaCl Concentration are as follows:

- Sodium chloride solutions are much less aggressive to cement paste in comparison to calcium/magnesium chlorides.
- Addition of sodium chloride converts AFm phases to Friedel salt, as is clearly observable by its (006), (0012), (018), (110) and (1112) reflections happening at 11.36°, 22.84°, 23.53°, 31.23°, and 39.04° angles, respectively.

$$Friedel\;salt\;formation:\;{2Cl}^{-}+2{\left[{Ca}_{2}•Al{\left(OH\right)}_{6}\right]}^{+}•({OH}^{-},{{CO}_{3}}^{-2},SO_{4}^{-2})•6H_{2}O\to 2{\left[{Ca}_{2}•Al{\left(OH\right)}_{6}\right]}^{+}•2{Cl}^{-}•2H_{2}O+({OH}^{-},{{CO}_{3}}^{-2},SO_{4}^{-2})+4H_{2}O$$

- Ettringite is relatively untouched; however, it signals display lessening signs at higher concentrations of NaCl, which might be due to partial reductions in the pore solution pH caused by buffering effect of sodium chloride.
- At the 5 M concentration, sodium chloride precipitates due to oversaturation and its (111), (200), (220), and (311) reflections appear at 27.47°, 31.82°, 45.62°, and 54.08° angles, respectively.

### 3.4. Influence of Low Temperature on the Reaction of Deicing Salts

Since deicing salts are usually deployed at low temperatures, the question arises whether the mechanism of interaction between the concrete pastes containing slag and deicing salt solutions is affected with temperature. If these reactions are thermodynamically favored, they might overcome the lower kinetics limitations imposed by lower temperatures. To better address this question, the results of experiments at ambient temperature are compared to the results of the experiment performed at low temperatures. The low temperature tests were performed by first applying the deicing salt solution to the ground slag + cement paste at room temperature and then immediately keeping the samples at −18 °C for more than three weeks and then testing them.

#### 3.4.1. Calcium Chloride

**Figure 8:**
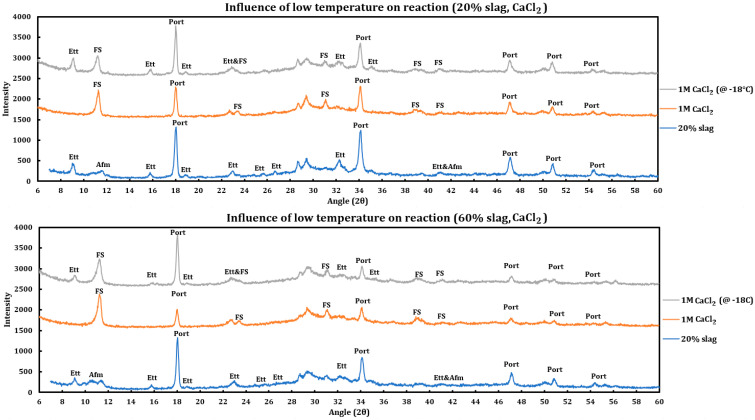
Influence of temperature (−18 °C) on the reaction of 1 M calcium chloride solution with slag + cement paste at two different slag replacements (20% and 60%).

To study the influence of low temperature on the reaction between deicing salts and slag + cement pastes, the samples containing 20% and 60% slag were reacted with 1 M CaCl_2_ and then left inside a freezer (~−18 °C) for three weeks. Afterwards, they were taken out of the freezer and tested with XRD immediately. Then, the resulting spectrum was compared to the spectrum related to the reaction of the deicing salt at ambient condition as well as the spectrum of the unreacted slag sample (Figure 8).

Influence of temperature on the reaction with 1 M CaCl_2_:

- Friedel salt has been formed in both cases.
- Intensity of other reactions for the case of frozen samples is lower than the samples reacted at ambient temperature, which is a sign that the reactions were not thermodynamically favored at lower temperatures. The corroborating evidence is as follows:
- While both cases displayed reaction of portlandite, the intensity of portlandite dissolution is higher in the case that has reacted at ambient temperature.
- Ettringite has not reacted noticeably in the case that has reacted in freezer while it has been almost depleted in the case that has been reacted at ambient temperature.
- The same trend is observed in both cases (20% and 60% slag replacement). Therefore, the same issue is expected regardless of the amount of slag replacement.

#### 3.4.2. Magnesium Chloride

**Figure 9:**
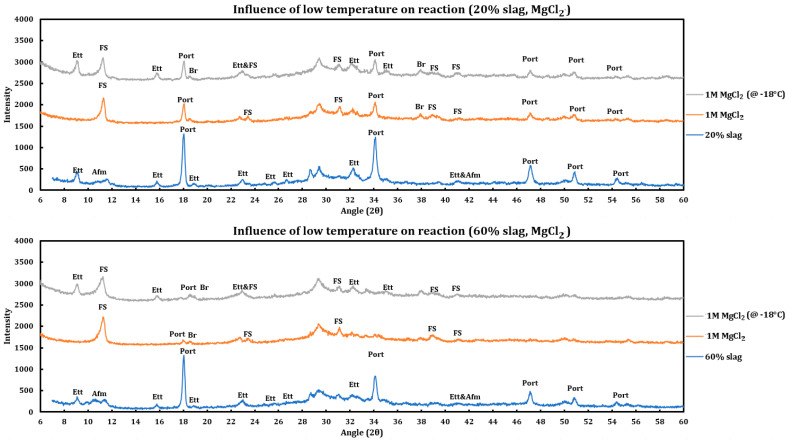
Influence of temperature (−18 °C) on the reaction of 1 M magnesium chloride solution with slag + cement paste at two different slag replacements (20% and 60%).

Similar to the CaCl_2_ case, experiments have been performed to check the influence of temperature on the reaction of 1 M MgCl_2_ with slag + cement pastes, and the results resemble the previous results, which are displayed in Figure 9 and are as follows:

- Friedel salt has been formed in both cases.
- Portlandite has been depleted in both cases.
- The extent of the reaction was lower for the case stored in the freezer, as the ettringite has not reacted noticeably for this case, while it has been almost depleted in the case that has been reacted at ambient temperature.
- The same trend is observed in both cases (20% and 60% slag replacement). Therefore, the same conclusion stands regardless of the amount of slag replacement.

#### 3.4.3. Sodium Chloride

**Figure 10:**
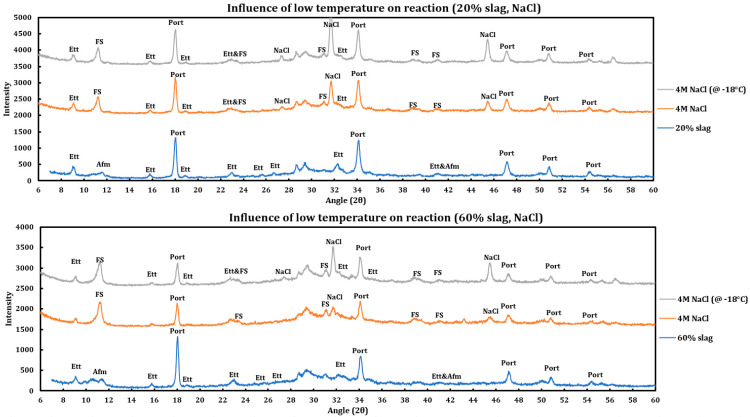
Influence of temperature (−18 °C) on the reaction of 4 M calcium sodium solution with slag + cement paste at two different slag replacements (20% and 60%).

Influence of temperature on the reaction of slag + cement paste with 4 M NaCl has been studied (Figure 10) and the results are as follows:

- The reaction products are almost identical in both cases.
- Friedel salt has been formed in both cases.
- Portlandite or ettringite have not reacted in any of the cases.
- The same trend is observed in both cases (20% and 60% slag replacement). Therefore, the same issue is observable regardless of the amount of slag replacement.

### 3.5. Effect of Slag Replacement

**Figure 11:**
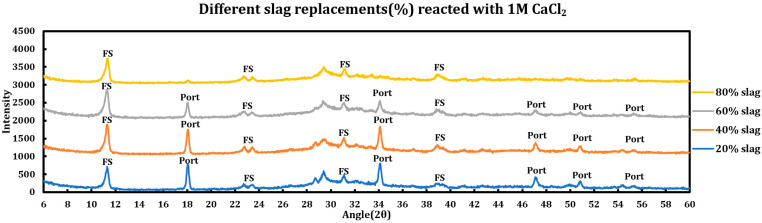
Influence of slag replacement on the extent of the reaction between 1 M calcium chloride solution and slag + cement pastes.

**Figure 12:**
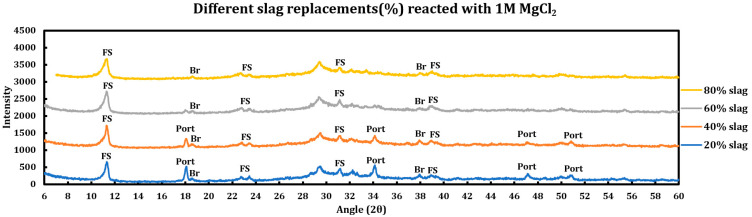
Influence of slag replacement on the extent of the reaction between 1 M magnesium chloride solution and slag + cement pastes.

**Figure 13:**
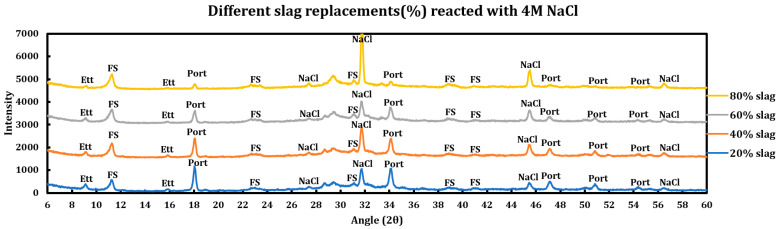
Influence of slag replacement on the extent of the reaction between 4 M sodium chloride solution and slag + cement pastes.

Influence of slag replacement on the extent of the chemical deterioration induced by deicing salts on slag + cement pastes was also investigated, and the results are displayed in the Figure 11 (CaCl_2_), Figure 12 (MgCl_2_), and Figure 13 (NaCl). Increasing slag replacement levels will concentrate some phases at the expense of other phases and this issue might have implications for the extent of the chemical corrosion caused by deicing salt solutions. During these experiments, the concentration of the deicing salt solution is kept constant while the slag replacement increases from 20% to 80%.

#### 3.5.1. Calcium Chloride (1 M CaCl_2_)

Influence of slag replacement on the reaction of slag + cement paste and 1 M calcium chloride solution is as follows:

- Portlandite depletion: at higher levels of slag replacements, portlandite has been depleted more quickly and particularly in the case of 80% slag, it is not noticeable after the reaction.
- Since at higher levels of slag replacement, more AFm phases are formed, accordingly, after the reaction with deicing salt, more Friedel salt is also observed at higher slag replacements.
- Ettringite has disappeared in all phases.

#### 3.5.2. Magnesium Chloride (1 M MgCl_2_)

Influence of slag replacement on the reaction of slag + cement paste with 1 M magnesium chloride solution is as follows:

- Intensity of the portlandite signals is lower in comparison to samples reacted with CaCl_2_ and completely disappeared at the 60% slag replacement. This issue is more due to conversion of portlandite to brucite in the solution containing magnesium chloride.
- Ettringite has disappeared in all phases.

#### 3.5.3. Sodium Chloride (4 M NaCl)

Influence of slag replacement on the reaction of slag + cement paste with 4 M sodium chloride solution is as follows:

- The reduction in portlandite content is only due to pozzolanic reactions and not the reaction with deicing salts.
- Ettringite is clearly observed in all cases; however, it is observed with a lower intensity at higher slag replacements due to the dilution effect of SCMs.
- Intensity of FS has increased at higher slag replacements, which might have originated from the extra AFm phases produced by the higher incorporation of slag.

### 3.6. Solubility of Friedel Salt in Deicing Salt Solutions

**Figure 14:**
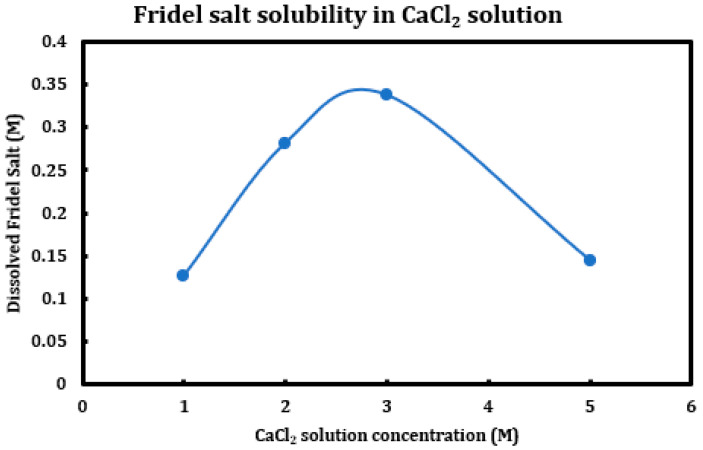
Solubility of Friedel salt at different concentrations of calcium chloride solutions.

**Figure 15:**
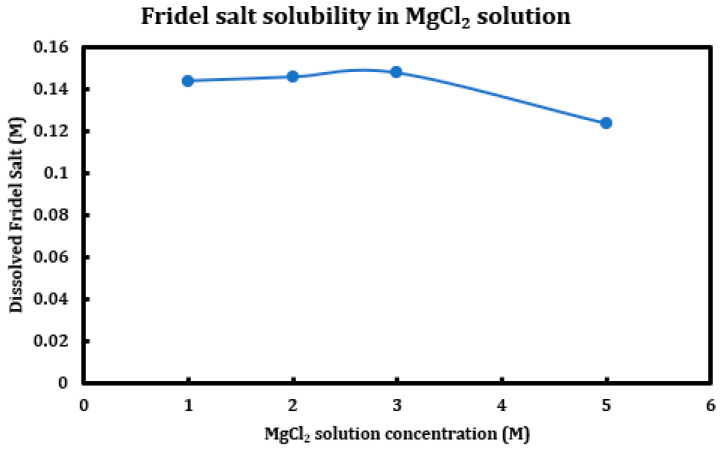
Solubility of Friedel salt at different concentrations of magnesium chloride solutions.

**Figure 16:**
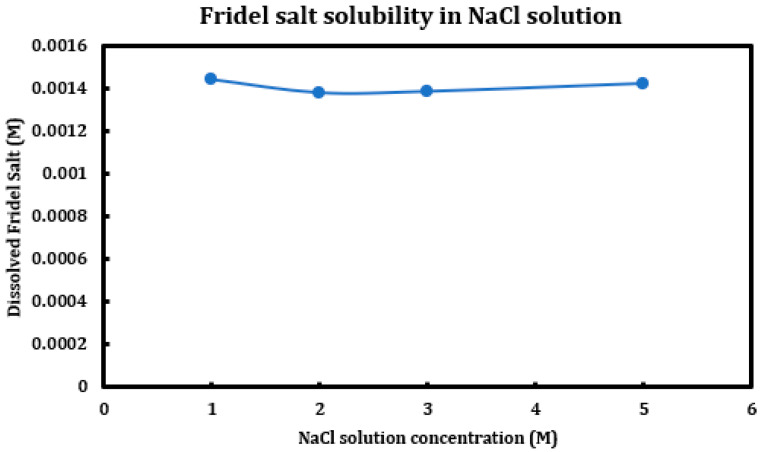
Solubility of Friedel salt at different concentrations of sodium chloride solutions.

During a previous phase of this study, it was found that formation and later dissolution of the Friedel salt in slag + cement pastes provides a route for the deterioration of the resulting concrete paste. Here, in this phase, we have tried to quantify this phenomenon. Therefore, it was necessary to synthesize Friedel salt and to react it with various deicing salt solutions (NaCl, MgCl_2_, CaCl_2_) at different concentrations (1 M, 2 M, 3 M, and 5 M) and then provide the reaction with a sufficient amount of time and continuous mixing for 30 days to reach equilibrium. Mixing was then stopped and after 30 days, the separated solution was extracted using syringe filters and after dilution and mixing with nitric acid (0.2 M), it was tested using ICP-OES. Based on stoichiometry, it was possible to measure the amount of Friedel salt dissolved into the solution. Later on, the solid residue left after dissolution was also filtered, washed, dried, and characterized using XRD. The results are presented in Figure 14 (CaCl_2_), Figure 15 (MgCl_2_), and Figure 16 (NaCl).

The results clearly indicate the correlation between the solution pH and the solubility of the Friedel salt in the system. Increasing concentration of calcium/magnesium chloride in the system clearly reduces the system pH. When concentration of CaCl_2_ increases from 1 M to 3 M, there is a clear increase in the solubility of the Friedel salt in the system. This increase in the solubility is in line with the increase in the system pH. However, as the concentration of CaCl_2_ increases and reaches 5 M, the solubility shows clear signs of decline and becomes closer to the solubility when the concentration was 1 M. The reason for this reduction in solubility might be ascribed to the presence of common ions and reversal of the equilibrium based on the Le Chatelier’s principle. Since dissolution of Friedel salt is incongruent and liberates 4Ca^+2^ and 2Cl^−^ ions per molecule of Friedel salt, higher CaCl_2_ concentrations might therefore reverse the dissolution of this salt. Similarly, magnesium chloride dissolution in water reduces its pH and also solubility of Friedel salt is similarly considerable; however, the rate of increase and its ensuing reduction in the solubility are less intense than the case of calcium chloride solutions.

On the other hand, the solubility of Friedel salt is considerably less in the case of sodium chloride solutions in contrast to the calcium/magnesium chloride solutions. As is observed, the pH of the solution is relatively less touched in the case of sodium chloride solutions and therefore its solubility of Friedel salt. Based on the measured quantities, the solubility of Friedel salt in calcium/magnesium chloride solutions is more than two orders of magnitude higher than its solubility in the sodium chloride solution. This issue clearly undermines stability of all AFm phases that are in contact with deicing salt solutions containing calcium/magnesium chloride.

The solid residues were characterized as discussed below.

The incongruent dissolution mechanism for Friedel salt is as follows:

$$2{\left[{Ca}_{2}•Al{\left(OH\right)}_{6}\right]}^{+}•2{Cl}^{-}•2H_{2}O\to {2Cl}^{-}+{4Ca}^{+2}+2Al{\left(OH\right)}_{3}↓+6{\left(OH\right)}^{-}+2H_{2}O$$

**Figure 17:**
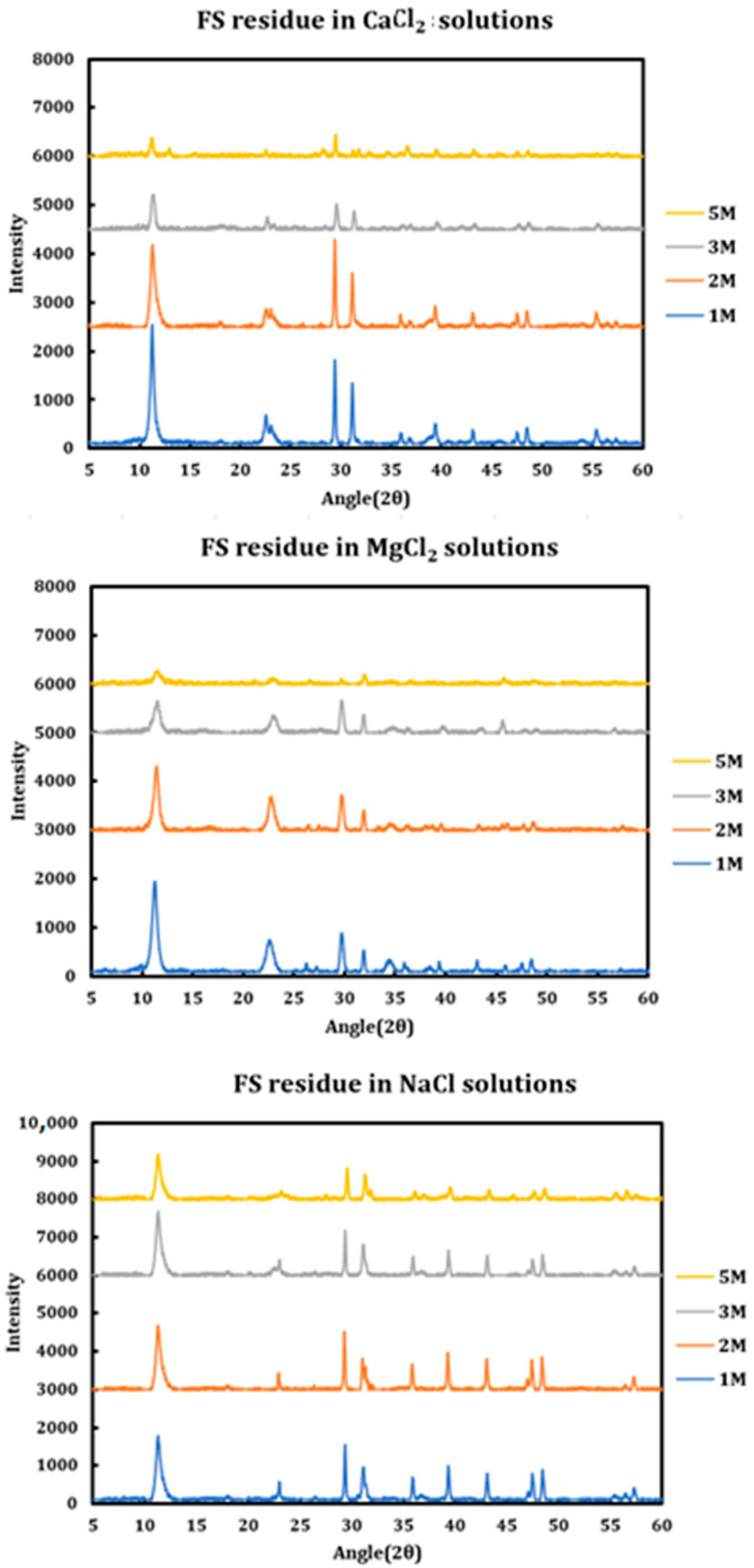
XRD spectra of the solid residue after dissolution of Friedel salt in deicing salt solutions.

The aluminum hydroxide formed on the other side of the reaction has an amorphous form and therefore does not have reflections in the resulting XRD spectra. The major crystalline phase shown by XRD spectra is the Friedel salt diluted by amorphous aluminum hydroxide which has been subtracted from the main spectra as the background. The XRD spectra of the remaining residue is as illustrated in Figure 17 below.

## 4. Discussion

### 4.1. Oxychlorides vs. Friedel Salt

Despite the fact that some of the literature that considers FS only when sodium chloride is used as a deicing salt [48], FS is a common product when cementitious phases are contacted with chloride containing solutions regardless of the cation that is accompanying the chloride ion. The chloride ion, with its high mobility, can penetrate deep into the cement matrix and form FS either through surface adsorption on calcium aluminate hydroxide cations or via ion exchange and replacement of the ions in the AFm phases. Probably one of the reasons that some researchers have not identified FS in their system was the high concentration of deicing salt which decomposed FS during the reaction period.

Calcium oxychloride, as reported by Collepardi [49], is not present in the system. Although its 10.62° reflection becomes entrapped under the corresponding FS signal, the strongest reflection that is reported for this phase is anticipated to occur at 21.5°. while this signal is absent in all derived spectra. On the other hand, the X-ray diffraction spectrum of COX as reported by Collepardi does not seem to be even close to the spectrum reported for this phase in the Joint Committee on Powder Diffraction Standards (JCPDS) Card No. 02-0280. However, the dried COX [19,50] ($Ca{Cl}_{2}\cdot Ca{\left(OH\right)}_{2}\cdot 2H_{2}O$) has clearly distinct peaks at 18.04°, 28.33°, 32.56°, and 38.43° that can show the presence of dried COX at higher calcium chloride concentrations.

Addition of slag to the concrete paste mixture will enhance the ratio of AFm phases to portlandite in contrast to a concrete paste made up of pure cement. Portlandite is consumed not only via the pozzolanic reaction but also through its reaction with the amorphous alumina supplied by slag as a result of which further AFm phases form in the system. Therefore, while there is not much portlandite in the system to produce plenty of oxychlorides, AFm phases will be formed in larger proportions and will convert to Friedel salt upon infiltration of the system with chloride ions and this issue is present regardless of the type of the cation that is accompanying the chloride ion, and in any case, FS will be formed.

Accordingly, FS has been shown to be more chemically corrosive to concrete paste than oxychlorides. While COX has been demonstrated to remain in the system even when the calcium chloride concentration is as high as 5 M, stability of FS was limited, and it dissolved incongruently in the pore solution as the concentration of the calcium/magnesium chloride increased. The calcium ions that are liberated during this incongruent dissolution can react with chloride ions and water and form calcium oxychloride crystals, as formation of oxychlorides has been demonstrated to be possible from their building blocks [51]. This finding is also in line with the literature, as COX/MOX were observed at higher concentrations of the related salts [52]. On the other hand, the COX crystals after dehydration seem to be stable and remain in the system; therefore, they can be considered as less corrosive.

Probably one of the reasons that oxychlorides have received wider attention in contrast to FS is that most researchers probing into the corrosive effect of deicing salts on concrete pastes have been looking for what is present in the system, while part of the answer might be related to the species that are not present in the system and by their dissolution have increased the permeability of the system and enabled the aggressive ions to reach deep into the concrete paste.

### 4.2. Influence of Design Parameters

Influence of various design parameters such as type, concentration, temperature, and slag replacement levels have been studied during this research. Influence of the type of deicing salt was considered during this study, and it was found that the type of the deicing salt used has a substantial effect on the mechanism and the extent of the chemical deterioration occurred. While sodium chloride converts the AFm phases to Friedel salt, its effect is almost limited to that extent, as the other phases in the system are relatively untouched. On the other hand, calcium/magnesium chloride display a much more corrosive effect on the concrete pastes made up of the mixture of slag + cement. The solutions of these salts can not only convert all AFm phases to Friedel salt but also can dissolve it and decompose ettringite and initiate different phenomena at different concentrations. Despite previous findings [53], gypsum was not formed during ettringite decomposition. The mechanism during which ettringite is converted to FS is not clear for the author. There is a possibility that ettringite first decomposes to its building blocks and then they in return contribute to the formation of FS. The effect of the concentration of the deicing salts was also analogous. Higher concentrations of sodium chloride did not result in other phenomena while behavior of calcium/magnesium chloride was affected by their concentration. At lower concentrations, ettringite was decomposed, but at higher concentrations it formed again and then finally decomposed. Friedel salt was formed considerably at lower concentrations, but it started to dissolve as the concentration increased. Calcium/magnesium oxychlorides were formed at relatively higher concentrations.

Influence of temperature was studied separately, and it was found that low temperatures just reduce the kinetics of the reaction without thermodynamically favoring it. As a result, the chemical reaction between deicing salt solutions and slag + cement paste is just decelerated at lower temperatures and the fact that higher damages might be observed at low temperatures is more related to the physical phenomena such as freeze/thaw that are usually concomitant with this phenomenon. As the slag replacement levels increase in the slag + cement pastes, the amount of the portlandite produced decreases and as a result, portlandite depletion occurs faster when the paste is contacted with calcium/magnesium chloride solutions; similarly, calcium /magnesium oxychloride formation also happens to a much lesser extent.

Contrary to some previous studies reporting that magnesium chloride is considerably more chemically corrosive to concrete paste in comparison to calcium chloride, in this study, it has been observed that the extent of their corrosiveness is similar. Considering the fact that calcium chloride is implemented in higher concentrations in contrast to magnesium chloride, the extent of their chemical attack is thought to be close.

### 4.3. Solubility of FS in Deicing Salt Solutions

Friedel salt, as one of the main suspects causing chemical deterioration in slag + cement paste, was synthesized using wet chemistry matter and after washing and drying, it was contacted to different concentrations of calcium/magnesium/sodium chloride solutions. The suspension was provided with an ample time of one month, after which the liquid part was extracted, diluted, and tested with ICP-OES for the concentrations of calcium ion, through which the solubility of Friedel salt in different deicing salt solutions was calculated. It was found that that the solution pH had a significant effect on the solubility of this salt, as its solubility in calcium/magnesium chloride solutions is notably higher than its value in sodium chloride. The trend of changes in solubility of FS versus concentration of the deicing salt is not uniform among different deicing salt solutions.

### 4.4. Dichotomy in the Literature Regarding the Role of SCM on Resistance Against Deicing Salts

There is a kind of disagreement in the literature regarding the influence of slag incorporation on the resistance of the resulting concrete paste against deicing salt solutions. While some articles argue that slag replacement will enhance this attribute [18,54], others contend that addition of this SCM will cause vulnerability against these media [21,22,26]. What we understand from this research coupled with previous studies is that while presence of slag in the concrete matrix will result in a concrete with superior physical properties such as higher compressive strength and better pore structure, its paste will develop some vulnerable points that can show themselves if the contact time between the attacking species and the paste is sufficient. As a result, while addition of slag to concrete will improve its physical resistance against deicing salt solutions [55], some features of its chemical resistance against such materials is compromised. Therefore, depending on the type and characteristics of the experiment performed, the physical or the chemical attribute of this interaction will become more pronounced. Since the rate of the chemical reaction is not high, some considerations are needed to capture this feature of the phenomenon in the lab. For instance, in this study, slag + cement paste was fine ground and then reacted with deicing salts at different concentrations and given sufficient time to enable occurrence of the chemical reactions.

Normally, most related tests are performed on concrete samples and have practical time limitations as they cannot take infinite time. Thence, not only presence of aggregates in the concrete matrix will slow down the diffusion of the chemicals and restrict the extent of the chemical reactions accordingly, but also the limited times to perform such tests impose further hindering on the completion of the chemical reactions. However, concrete structures in nature do not face the latter restriction and the chemical reaction will take place sooner or later.

Concretes containing slag might show higher resistance against deicing salts because attacking ions have a harder time penetrating these concretes thanks to their lower permeability, and after infiltration and interactions, these ions face more difficulties damaging the concrete since these concretes have a higher mechanical strength. However, its paste has its own implications and weak points that will show themselves sooner or later.

### 4.5. Caveats in Assumptions Related to Chloride Binding

It has been assumed that Friedel salt can enhance the chloride binding capacity of the concrete and therefore guard the rebar against the chloride corrosion, but this study can provide some clues, as the chloride binding of the FS is not permanent in all conditions, as the stability of this salt is seriously under question at low pH conditions; therefore, as the pH of the system falls below some limits, FS will become decomposed and all of the bound chloride will be released to the system and attack the rebar.

In the end, it is necessary to consider that the current manuscript aims at a better understanding of the chemical reactions between slag-containing pastes and deicing salts. When considering the concrete as a whole, it is necessary to consider other influencing factors as the recent studies have shown that incorporating slag cement impacts the concrete’s microstructure and therefore resistance to chloride ingress under deicing salt and freeze/thaw conditions [56,57,58,59]. Simultaneously, factors such as curing quality, slag fineness, carbonation, and binder composition significantly affect that criterion [60,61,62]. Ultrafine and alkali-activated slags further improve resistance, though they remain sensitive to environmental conditions [57,60,63]. Scaling and deterioration may intensify under combined chloride and freeze/thaw exposure due to ice formation, cryogenic suction, and chloride binding—with slag mixes behaving differently than conventional concretes [57,62,64]. These findings highlight the various aspects related to the durability of concretes made up of slag cement in cold, salt-exposed environments.

## 5. Conclusions

Based on the data collected and observations, we can conclude the following:

- Addition of slag cement to concrete paste leads to two contrasting effects on the resistance of concrete against deicing salt solutions. While slag incorporation can increase the mechanical strength and reduce the portlandite available for oxychloride formation and therefore increase the resistance against deicing solutions, there is an accompanying disadvantage. Addition of slag to concrete paste can enhance the quantity of the AFm phases in the concrete, which will convert to Friedel salt upon reaction with chloride-based deicing solutions. This salt has low stability in acidic environments caused by calcium/magnesium chloride solutions and dissolves in these solutions and leaves voids in the concrete matrix.
- Dissolution of Friedel salt (FS) in lower pH conditions is considered to be a more corrosive issue than oxychloride formation. Although oxychloride is formed chemically, its impact is more a physical effect through its volume expansion. It seems to be stable in environments with a low pH. On the other hand, FS dissolves in low pH environments and increases porosity.
- At higher slag replacements, more AFm phases are formed and therefore more FS is produced when the concrete is placed in contact with solutions containing chloride ions.
- Calcium/magnesium chloride solutions are much more chemically corrosive to concrete paste in contrast to the sodium chloride solutions. Reduction in the pH in the pore solutions is considered to be a major contributing factor to this type of chemical deterioration.
- Influence of deicing salt solutions is not uniform at different concentrations. While some phases decompose at lower concentrations, they might reappear at higher concentrations and then again decompose.
- The chemical reaction between deicing salts with concrete paste will take place regardless of the temperature; however, the extent of the reaction is more pronounced in warmer conditions in contrast to cold weather.
- Solubility of the Friedel salt has been measured in calcium/magnesium/sodium chloride solutions for long mixing periods (30 days) to ensure equilibrium and it has been observed that while FS is relatively stable in sodium chloride solutions, its solubility in calcium/magnesium chloride solutions can become considerable.
- In some articles, it has been assumed that concretes containing slag have better resistance against chloride solutions thanks to their higher chloride binding. It needs to be considered that the bound chloride can become released if the pH of the pore solution falls locally. Conversion of AFm phases to the Friedel salt is one of the main contributors to the chloride binding of concrete and while this salt is stable in basic and neutral conditions, it decomposes in acidic conditions, and the previously bound chloride will be released.
- This manuscript only studied the impact of chloride-based deicing salts on the concretes containing slag. However, there are other types of deicing salts, and their influence can be studied separately. This can also be the future direction of studies in which impact of other types of deicing salts are studied and potential deicing solutions that are less detrimental to the slag-containing concretes are devised.

## Figures and Tables

**Table 1:** Chemical composition (elemental analysis) of the cement and slag used in the experiments.

Oxides	Cement (wt%)	Slag (wt%)
CaO	68.81%	44.66%
$SiO_{2}$	17.42%	31.56%
${Al}_{2}O_{3}$	4.54%	9.37%
${Fe}_{2}O_{3}$	3.35%	0.74%
MgO	1.25%	9.04%
$K_{2}O$	0.84%	1.03%
${Na}_{2}O$	0.13 %	0.29%
$SO_{3}$	3.77%	2.86%
$TiO_{2}$	0.02%	0.44%
MnO	0.02%	0.01%
$P_{2}O_{5}$	0.01%	0.01%

**Table 2:** Samples reacted with deicing salt solutions at different conditions.

Slag Replacement (wt%)	CaCl_2_ Concentration	MgCl_2_ Concentration	NaCl Concentration	Temperature
20	1 M	1 M	2 M, 4 M	20 °C, −18 °C
40	1 M, 2 M, 3 M, 5 M	1 M, 2 M, 3 M, 5 M	2 M, 3 M, 4 M, 5 M	20 °C
60	1 M	1 M	4 M	20 °C, −18 °C
80	1 M	1 M	4 M	20 °C

**Table 3:** Solubility of Friedel salt is measured in the dicing salt solutions with the following conditions.

CaCl_2_ Concentration	MgCl_2_ Concentration	NaCl Concentration
1 M	1 M	2 M
2 M	2 M	3 M
3 M	3 M	4 M
5 M	5 M	5 M

## Data Availability

The original contributions presented in this study are included in the article. Further inquiries can be directed to the corresponding authors.

## Glossary

AFm	Alumina, Ferric oxide, Monosulfate phase
Bel	Belite
Bru	Brucite
COX	Calcium Oxychloride
F/T	Freeze/Thaw
Fer	Ferrite
FHS	Siliceous Hydrogarnet
FS	Friedel’s Salt
Geo	Gorgeyite
HT	Hydrotalcite
ICP-OES	Inductively Coupled Plasma Optical Emission Spectroscopy
LDH	Layered Double Hydroxide
MOX	Magnesium Oxychloride
OPC	Ordinary Portland Cement
Port	Portlandite
SCM	Supplementary Cementitious Materials
Syn	Syngenite
XRD	X-Ray Diffraction
XRF	X-Ray Fluorescence
